# Distanzelektroimpulsgeräte (DEIG)

**DOI:** 10.1007/s00399-024-01049-3

**Published:** 2024-11-11

**Authors:** Jana Ackmann, Daniel Steven

**Affiliations:** Klinik III für Innere Medizin, Abteilung für Elektrophysiologie, Universität zu Köln, Medizinische Fakultät und Uniklinik Köln, Kerpener Str. 62, 50937 Köln, Deutschland

**Keywords:** Taser, Herzrhythmusstörung, Kammerflimmern, Polizei, Elektroschock, Taser, Cardiac arrhythmia, Ventricular fibrillation, Police, Electroshock

## Abstract

**Hintergrund:**

Distanzelektroimpulsgeräte (DEIG, „Taser“) werden zunehmend auch in Deutschland eingesetzt. Sie sollen durch die Androhung des Einsatzes die Gewaltdelikte gegenüber Polizeibeamten verhindern und durch den ggf. notwendigen Einsatz mittlere und schwere Verletzungsfolgen bei Beamten und gewalttätigen Störern reduzieren. Durch die Abgabe repetitiver Stromimpulse kommt es zur neuromuskulären Lähmung und Handlungsunfähigkeit. Es gibt jedoch Sicherheitsbedenken aufgrund von Todesfällen im Zusammenhang mit DEIG.

**Ziel der Arbeit:**

Ziel der Arbeit ist es, einen Überblick über mögliche medizinische Risiken des Einsatzes von DEIG aufzuzeigen.

**Methoden:**

Es erfolgte eine ausführliche Literaturrecherche in relevanten Datenbanken.

**Ergebnisse:**

DEIG bieten insgesamt ein gutes Sicherheitsprofil, können aber selten schwere oder lebensbedrohliche gesundheitliche Folgen haben. Hierzu gehören Verletzungen vulnerabler Körperregionen und Sturzfolgen. Ein myokardiales Capture und maligne Arrhythmien sind im Tierexperiment auslösbar. Die Kausalität von Todesfälle nach DEIG-Einsatz kann bei konkurrierenden Ursachen meist nicht mehr eindeutig zugeordnet werden, in den vorhandenen Studien mit Freiwilligen konnten bisher aber keine Arrhythmien beobachtet werden. Insgesamt scheint das Risiko für lebensbedrohliche Herzrhythmusstörungen beim Menschen gering, wenn auch vorhanden, zu sein. Zu den besonders gefährdeten Risikogruppen gehören Kinder, psychisch Vorerkrankte, Intoxikierte, Schwangere und Device-Träger.

**Fazit:**

Auch wenn DEIG prinzipiell maligne Arrhythmien auslösen können, ist das Risiko gering und die Kausalität von Todesfällen nach DEIG-Einsatz oft nicht sicher zu klären. In der medizinischen Versorgung von Patienten nach DEIG-Einsatz sind vor allem Komorbiditäten wie Intoxikationen oder psychische Erkrankungen zu bedenken.

## Abstract (en)

**Background:**

Conducted energy weapons (CEW, TASER) are increasingly used by law enforcement agencies in Germany. The CEWs are intended to reduce violence against police officers and to reduce moderate to severe injuries of police officers and aggressors. The delivery of repetitive electrical impulses causes neuromuscular paralysis and incapacitation; however, there are safety concerns due to deaths related to CEWs.

**Objective:**

The aim of this article is to provide an overview of the medical risks associated with the use of CEWs.

**Methods:**

A literature search was carried out using relevant databases.

**Results:**

The CEWs offer a good overall safety profile but in rare cases can have serious or life-threatening health risks. These include injuries to vulnerable body regions and falls. Myocardial capture and malignant arrhythmia can be triggered in animal experiments. The cause of death following the use of a conducted energy device (CED) can often not be clearly attributed due to competing causes; however, in studies with volunteers arrhythmia has not been observed so far. Overall, the risk of life-threatening cardiac arrhythmia in humans appears to be low, although still present. Particularly vulnerable risk groups include children, people with a history of mental illness or with intoxication, pregnant women and cardiac device wearers.

**Conclusion:**

Even though in principle CEWs can trigger malignant arrhythmia, the risk is low and the causality of death after CEW deployment often remains unclear. In the medical care of patients after CEW use, comorbidities such as intoxication or mental illness must be considered.

## Technologie von Distanzelektroimpulsgeräten

Distanzelektroimpulsgeräte (DEIG), umgangssprachlich Taser, werden international und in Teilen Deutschlands zunehmend im Polizeidienst eingesetzt. Die Geräte können eine neuromuskuläre Lähmung und Handlungsunfähigkeit herbeiführen. Auch sollen sie sichtbar getragen eine abschreckende Wirkung haben. Jedoch bringt der Gebrauch auch die Sorge von medizinischen Risiken mit sich. Es gab einige Todesfälle in zeitlicher Nähe zum Einsatz von DEIG, deren Kausalität nicht klar ist und die kontrovers diskutiert werden, insbesondere im Hinblick auf die Auslösung maligner Herzrhythmusstörungen.

Das erste DEIG wurde in den 1960er-Jahren von Jack Clover erfunden [19]. Nach technischer Modifikation wurden diverse kommerziell erhältliche Modelle produziert. Der Taser X26E (Axon Enterprise, Inc. Scottsdale, USA) ist das DEIG, zu dem die meisten Studien existieren und wurde bis 2014 vertrieben. Es folgten weitere Nachfolgemodelle wie 2011 der Taser X2, 2013 der Taser X26P, 2018 der Taser T7 und 2023 der Taser 10. Während das grundlegende Funktionsprinzip gleich ist, unterscheiden sich die Modelle in technischen Details.

DEIG können entweder im Kontakt- oder Distanzmodus eingesetzt werden. Im Kontaktmodus wird bei direktem Aufsetzen des DEIG auf die Haut Strom abgegeben, welcher zu einer akuten Schmerzreaktion führt. Die elektrischen Felder überschreiten die Schwelle zur Auslösung von Kammerflimmern oder kardialem Pacing nur in den subkutanen und kutanen Schichten, sodass systemische Effekte unwahrscheinlich sind [17]. Es wurden lokale Hautrötungen und teils auch Verbrennungen beobachtet. Im Distanzmodus hingegen werden mit komprimiertem Stickstoff Sonden durch das Gerät abgefeuert, deren Metallverankerungen Kleidung und Haut durchdringen. Die Sonden bleiben durch ummantelte Drähte mit dem DEIG verbunden. Zwischen ihnen werden Stromimpulse durch den Körper gesendet. Auch ein Hybridmodus mit direktem Aufsetzen des DEIG, nachdem die Sonden bereits abgegeben wurden, ist möglich.

Jede Auslösung setzt einen 5‑sekündigen Zyklus mit hochfrequenter Abgabe von Stromimpulsen in Gang, der durch Halten des Abzugs verlängert oder durch Betätigung des Sicherungsschalters abgebrochen werden kann [27]. Zu Beginn des Zyklus generieren die Geräte eine Leerlaufspannung von 50.000 V, die einen Lichtbogen durch Luft oder Kleidung schlagen soll, beispielsweise wenn die Sonden in der Kleidung stecken bleiben und nicht in den Körper eindringen. Anschließend folgen wellenförmige, kurze Impulse geringer Stromstärke und hoher Spannung. Die Modelle X2 und T7 haben eine Ladungsmessschaltung, sodass eine konstante Impulsladung mit geringen Schwankungen aufrechterhalten wird. Beim Taser X2 beträgt die effektive Stromstärke 1,2 mA und die maximale Spannung, die den Körper erreicht, 840–1440 V. Beim Taser T7 beträgt die effektive Stromstärke 1,3–1,5 mA und die maximale Spannung, die den Körper erreicht, 1500–2600 V [13].

Die Impulse setzen einen überschwelligen Reiz, der sowohl über eine direkte als auch eine indirekte Wirkung auf die Skelettmuskulatur eine Kontraktion bewirkt. Da vorwiegend Aα-Motoneurone stimuliert werden, fehlt die Möglichkeit, Willkürbewegungen auszuüben, und es resultiert die Handlungsunfähigkeit für die Dauer der Stromabgabe [11]. Das Funktionieren dieses Mechanismus hat zur Bedingung, dass die Elektrodenposition und der Abstand der Sonden voneinander adäquat ist und das Spannungsfeld auf eine ausreichende Menge Gewebe wirkt. Im Experiment ist hierfür ein Sondenabstand von etwa 23 cm notwendig [8]. Unter praktischen Bedingungen zeigte sich in einer großen Analyse aus Großbritannien der Taser X2 bei etwas mehr als zwei Drittel der Einsätze effektiv. Zu den häufigsten Gründen einer ineffektiven Wirkung zählten dicke und lockere Kleidung, das Verfehlen des Ziels mit den Sonden, aber auch ein zu geringer Sondenabstand. Hierbei schnitt ebenfalls ein Abstand der Sonden unter 23 cm schlechter ab [20].

**Abb. 1:**
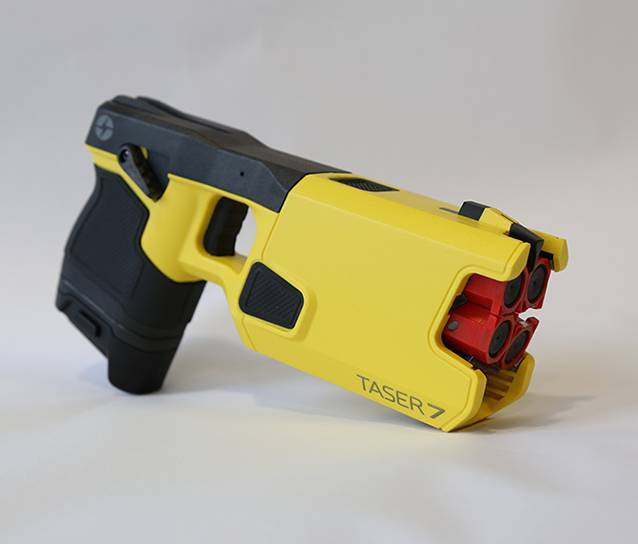
TASER 7. (Mit freundlicher Genehmigung des Landesamtes für zentrale polizeiliche Dienste NRW)

Die Modelle T7 und X2 besitzen im Gegensatz zu den Vorgängermodellen zwei Sondenpaare, sodass ein zweiter Schuss abgegeben werden kann. Der Taser T7 (Abb. 1) hat zudem eine „Cross-connect-Technologie“, sodass nach Abgabe von zwei Sondenpaaren der Stromfluss zwischen allen möglichen Vektoren adaptiert wird, um die Verbindung zu optimieren [13]. Das neueste Modell Taser 10 hat zehn einzelne Sonden, die nacheinander abgefeuert werden können.

## Medizinische Risiken

Mittlerweile gibt es diverse Tierexperimente, Fallberichte und kleine Studien mit Freiwilligen, die die Effekte von DEIG untersuchen. Gesundheitliche Risiken entstehen zum einen durch die Sonden und das Sturzrisiko und zum anderen durch die abgegebenen Stromimpulse.

### Mechanische Verletzungen durch Sonden und Sturzfolgen

Regelmäßig kommt es durch die Sonden zu leichten, lokalen Hautverletzungen wie oberflächlichen Verbrennungen und Schürfwunden. Bei Eintritt der Sonden in empfindliche Körperregionen wie Augen, Genitalbereich, Mund, Nacken und generell den Kopf können entsprechend weitere, potenziell schwerwiegendere, Verletzungen auftreten. Durch die neuromuskuläre Lähmung kann es außerdem zu einem ungebremsten Sturz kommen, sodass Kopfverletzungen zu den möglichen Folgen gehören. Diese reichen von Hämatomen bis hin zu schweren, lebensbedrohlichen intrakraniellen Blutungen [14]. Zur Abschätzung des Risikos analysierten Kroll et al. 2016 Todesfälle, die in Verbindung mit einer schwerwiegenden Kopfverletzung nach DEIG-Einsatz aufgetreten sind. Es wurden 16 Fälle identifiziert, in denen ein kausaler Zusammenhang bestand. Die Autoren folgerten, dass bei einer gesamten Anzahl von 3 Mio. DEIG-Nutzungen ein Risiko von 5,3 pro eine Million Einsätze für einen DEIG-induzierten Sturz mit Todesfolge bestünde [12].

### Kardiale Risiken und Todesfälle

Eines der wesentlichen Bedenken beim DEIG-Einsatz ist die Auslösung maligner Arrhythmien. In diversen Tierexperimenten am Schweinemodell konnten eine schnelle myokardiale Stimulation und teilweise auch eine Degeneration in maligne Arrhythmien wie Kammerflimmern beobachtet werden. Hierbei ist das Risiko abhängig von der Sondenposition, und mehrere Untersuchungen zeigten, dass das myokardiale Capture von herznahen Positionen und Eindringtiefe der Sonden abhing. Letztendlich zeigen sich die Abstände der Sonden zum Herzen, die ausreichend für die Induktion von Kammerflimmern waren, zwischen verschiedenen Studien am Tier variabel zwischen 6,2 und 100 mm [5, 26].

Zu den Hauptlimitationen von Tiermodellen gehören das häufig niedrigere Körpergewicht der verwendeten Schweine mit näherem Sondenabstand zum Herzen, unterschiedliche elektrophysiologische Eigenschaften des Schweineherzens und unrealistische experimentelle Bedingungen. Daher bestehen Zweifel, ob die Ergebnisse auf den Menschen übertragbar sind. In mehreren Studien am Menschen, die die Auswirkungen von DEIG an Freiwilligen untersuchten, konnten elektrokardiographisch und echokardiographisch bisher keine malignen Herzrhythmusstörungen nachgewiesen werden [25]. Es kam allerdings zu einem Fall von nachgewiesenem myokardialem Capture während der Entladung eines Prototypen eines DEIG, der dann nicht in der Form auf den Markt gebracht wurde [4]. Auch ein Fall, bei dem ein myokardiales Capture während des DEIG-Einsatzes in dem Speicher des Schrittmachers nachzuweisen war, ist beschrieben, wobei sich hieraus keine klinische Relevanz ergeben hat [3]. Insgesamt ist eine Auslösung maligner Herzrhythmusstörungen prinzipiell möglich, das Risiko in der Praxis aber sehr gering.

Todesfälle, die im zeitlichen Zusammenhang mit DEIG-Einsatz und nicht aufgrund eines Sturzes auftreten, sind retrospektiv häufig schwieriger zu bewerten, da in einer Vielzahl von Fällen weitere Faktoren wie Drogeneinfluss, psychischer Stress oder Vorerkrankungen eine Rolle spielen [21]. Anders als in Studien findet während des polizeilichen DEIG-Einsatzes kein Monitoring der Vitalparameter statt, was eine Bewertung weiter erheblich erschwert. In einer retrospektiven Analyse von Swerdlow et al. wurde der primäre Rhythmus bei Todesfällen in zeitlichem Zusammenhang mit DEIG-Exposition untersucht. Bei 56 von 118 Fällen lag eine Rhythmusdokumentation vor. Kammerflimmern wurde im EKG-Monitoring in 4 Fällen beobachtet. In 52 weiteren Fällen lag ein nicht defibrillierbarer Rhythmus vor. Es wurde nur bei einem Todesfall ein unmittelbar nach dem DEIG-Schock stattfindender Kollaps beobachtet, passend zu einer DEIG-induzierten Herzrhythmusstörung [22]. Dieses spricht dafür, dass DEIG-induzierte Herzrhythmusstörungen eine seltene Ursache von Todesfällen in zeitlicher Nähe zum DEIG-Einsatz sind. Dies unterstützt auch eine retrospektive Studie von Bozeman et al., wobei es bei insgesamt 1201 DEIG-Einsätzen zu 2 Todesfällen kam, die nicht auf den DEIG-Einsatz zurückgeführt wurden und zu keinem Todesfall durch Herzrhythmusstörungen [2]. Insgesamt muss anhand der Datenlage von einem geringen kardialen Todesrisiko durch den Einsatz von DEIG ausgegangen werden.

### Spezielle Risiken für Schrittmacher- und ICD-Träger

Eine besonders zu berücksichtigende Gruppe von gefährdeten Personen können solche mit implantierten aktiven elektronischen Geräten sein, da es durch DEIG-Einsatz zu elektromagnetischer Interferenz (EMI) kommen kann. Bei Herzschrittmachern besteht das Risiko, dass es zu einem ventrikulären Oversensing mit folgender Inhibition der ventrikulären Stimulation kommt. Bei kurzer Dauer der DEIG-Einsätze von 5 s ist damit zu rechnen, dass die Unterdrückung der Stimulation für diesen kurzen Zeitraum auch bei schrittmacherabhängigen Patienten keine relevanten gesundheitlichen Folgen hat. Im Falle von implantierbaren Defibrillatoren (ICD) ist eine Fehldetektion des DEIG-Impulses in Form von ventrikulären Tachykardien und Kammerflimmern durch den ICD denkbar, was dann zu einer inadäquaten ICD-Entladung führen kann. Auch hier ist die Wahrscheinlichkeit für ein solches Ereignis gering, da die Applikation der Stromimpulse durch das DEIG zeitlich limitiert ist.

Wie bereits erwähnt, gab es bereits einen Nachweis von myokardialem Capture in einem Schrittmacherholter ohne eine Relevanz für die Funktionsweise des Schrittmachers [3]. In einer weiteren Übersichtsarbeit von Vanga et al. wird die Annahme, dass kurze DEIG-Einsätze keine klinisch relevanten Folgen haben, unterstützt. Die Gruppe berichtet in einer Fallserie von 6 Patienten mit Schrittmachern und ICDs, bei denen ein DEIG eingesetzt wurde, über keine anhaltende Interferenz mit dem System, da die Einsätze in keinem der Fälle länger als 5 s durchgeführt wurden [23]. Ein weiterer Bericht behandelt ein atriales Oversensing eines DEIG-Impulses bei einem CRT, welches zum Mode-Switch führte, ohne klinische Folgen für den Patienten [18]. In der Literatur ist jedoch auch ein Fall einer inadäquaten ICD-Schockentladung nach repetitivem DEIG-Einsatz beschrieben. Hierbei verursachte ein erster Impuls von 5 s durch EMI eine Fehldetektion von Kammerflimmern und ein Laden des ICDs. Nach dem Ende des DEIG-Impulses wurde wieder ein Sinusrhythmus erkannt, sodass die Abbruchkriterien erfüllt waren und es nicht zur ICD-Auslösung kam. Ein kurz darauffolgender zweiter DEIG-Impuls führte jedoch zur Redetektion von Kammerflimmern und einem erneuten Laden des ICDs. Bei nachfolgender Sinustachykardie ohne Erfüllung der Abbruchkriterien kam es dann zu einer ICD-Schockentladung. Die Autoren analysierten verschiedene Herstellereinstellungen von ICDs und folgerten, dass der inadäquate Schock auch mit den programmierten Detektionsalgorithmen dieses ICDs zusammenhing, da bei anderer Programmierung der zweiten Schock nicht abgegeben worden wäre [1].

Grundsätzlich kann ein DEIG-Einsatz zu einer Tachykardie und zu einer Detektion des Stromimpulses durch den Schrittmacher oder ICD führen. Aufgrund des kurzen Impulses hat dies für den Patienten meist keine Konsequenz. Bei der Programmierung eines ICDs muss grundsätzlich darauf geachtet werden, dass inadäquate Schocks so gut wie möglich vermieden werden. EMI durch DEIG mit Devices ist möglich und kann zu atrialem und ventrikulärem Oversensing sowie abhängig von der Programmierung des Devices auch inadäquaten ICD-Entladungen führen. Insgesamt kann das zusätzliche Risiko für Träger von Schrittmachern und ICDs bei einem kurzen DEIG-Einsatz aber als gering eingestuft werden.

### Muskuläre Effekte

Im Extremfall kann eine exzessive körperliche Aktivität eine Rhabdomyolyse auslösen. Da es beim DEIG-Einsatz zur starken Muskelkontraktion für die Dauer des Stimulus kommt, bestehen auch dahingehende Sicherheitsbedenken. Es gibt einige Einzelfallberichte über eine Rhabdomyolyse im zeitlichen Zusammenhang mit einer DEIG-Exposition [6], wobei in allen dieser Fälle auch andere Faktoren wie beispielsweise Intoxikation oder exzessive körperliche Aktivität als ursächlich in Betracht kommen. Diverse Studien mit freiwilligen Probanden zeigen vor allem bei prolongierten DEIG-Auslösungen mit körperlicher Aktivität vergleichbare, milde laborchemische Veränderungen von CK, Laktat und/oder pH-Wert, ohne Rhabdomyolyse oder relevante Elektrolytverschiebungen [25]. Beispielsweise in einer Studie von Ho et. al. hatte die Fortsetzung einer körperlichen Aktivität laborchemisch den gleichen Einfluss auf Azidose und Laktatanstieg, wie eine DEIG-Auslösung im Anschluss an körperliche Aktivität [9].

Prinzipiell ist auch eine Lähmung der Atemmuskulatur mit der Gefahr einer Atemdepression und Apnoephasen denkbar. Die Mehrheit der Daten zeigt keine Unterbrechung der Atmung während der DEIG-Exposition. Es gibt jedoch auch eine Untersuchung des Taser T7, wobei 2 der 9 Teilnehmer während eines DEIG-Impulses mit 2 Sondenpaaren mit Position am Rücken keine eigenen Atemzüge zeigten [10]. Auch in einer Studie von Van Meenen et al. wurden 23 Freiwillige einem Taser X26 für 5 s ausgesetzt und währenddessen die Atmung überwacht. Hierbei kam es zur Unterdrückung des normalen Atemmusters und insbesondere zu einer deutlichen Beeinträchtigung der Inspiration trotz willkürlicher Atemanstrengung [24]. Dieses würde praktisch bei kurzem DEIG-Einsatz von einigen Sekunden nicht ins Gewicht fallen, da derartig kurze Apnoephasen nicht zu einer relevanten Hypoxie führen.

## DEIG-Einsatz bei vulnerablen Gruppen

Etwa ein Viertel der Menschen, die einem DEIG ausgesetzt sind, sind Menschen mit psychischer Belastung, sodass diese vulnerable Gruppe überproportional häufig betroffen ist [7]. Weiterhin gibt es einen relevanten Anteil von Personen, der beim DEIG-Einsatz intoxikiert ist [21].

Trotz hohem Anteil an der Gruppe, die DEIG ausgesetzt ist, gibt es kaum Untersuchungen zu den Folgen für ebendiese Menschen. Hierbei ist ein höheres Risiko denkbar, beispielsweise durch die starke autonome Erregung, die Einnahme von Stimulanzien, Antipsychotika und Antidepressiva, die bereits allein für Herzrhythmusstörungen prädisponieren könnten sowie überproportional häufig auftretende somatische Begleiterkrankungen in dieser Gruppe [16]. Häufig wird in Studien von Patienten mit hohem psychischem Stress oder in einem „Mental-Health-Notfall“ [16] berichtet, ohne dass die detaillierte medizinische und psychiatrische Vorgeschichte dieser Personen bekannt ist. In einer Analyse von Strote et al. von Todesfällen, die in Zusammenhang mit DEIG-Einsätzen auftraten, zeigte sich in 75,7 % ein „excited delirium“, also ein starker psychischer Erregungszustand. In 78,3 % wurden toxikologisch Substanzen nachgewiesen, am häufigsten Kokain in 45,9 % der Fälle, jedoch auch in 8,1 % der Fälle Antipsychotika und in 10,8 % der Fälle Antidepressiva [21]. Allerdings handelt es sich hierbei um eine kleine Analyse von nur 37 Autopsiefällen. Repräsentative Erhebungen, wie viele der Personen, die einem DEIG ausgesetzt sind, tatsächlich Psychopharmaka einnehmen und ob diese bereits vor DEIG-Einsatz EKG-Veränderungen hatten, gibt es bisher nicht.

Auch Kinder sind durch geringes Körpergewicht und somit möglicherweise kleinerem Abstand der Sonden zum Herzen potenziell einer höheren Gefahr durch DEIG ausgesetzt. Eine mögliche Risikogruppe sind auch Schwangere. Ein Fall von einem Abort eine Woche nach DEIG-Exposition mit Sondenposition auf Abdomen und Bein wurde beschrieben, wobei der kausale Zusammenhang nicht geklärt ist [15].

## Fazit

Distanzelektroimpulsgeräte (DEIG) sind ein zunehmend eingesetztes Polizeimittel. Neben schwer zu bewertenden Fallberichten aus dem praktischen Einsatz gibt es einige Studien im Tierexperiment und einige kleine Studien mit wenigen Freiwilligen. Insgesamt ist die Datenlage jedoch nicht umfassend und es mangelt an großen randomisierten, kontrollierten Studien, die aus ethischen Gründen auch kaum durchführbar wären. Anhand der verfügbaren Daten und pathophysiologischen Mechanismen ist es prinzipiell möglich, dass Kammerflimmern durch DEIG unter passenden Umständen mit herznahen Sondenpositionen ausgelöst wird. Die Wahrscheinlichkeit hierfür erscheint aber unter praktischen Bedingungen beim Menschen gering, und bei den meisten Todesfällen, die im Zusammenhang mit einem DEIG-Einsatz aufgetreten sind, gibt es konkurrierende oder wahrscheinlichere Ursachen. Für bestimmte vulnerable Gruppen gibt es möglicherweise ein höheres Risiko, jedoch ist die Datenlage bisher sehr schwach.

### Key Points für die Praxis.

- DEIG sind ein weltweit zunehmend eingesetzte Polizeimittel.
- Relevante Verletzungen können durch Sonden und Stürze hervorgerufen werden.
- Eine schnelle myokardiale Stimulation mit Auslösung maligner Arrhythmien ist möglich, das Risiko ist in der Praxis aber sehr gering.
- Elektromagnetische Interferenz mit Devices ist möglich, hat bei kurzer DEIG-Anwendung aber meist keine klinische Konsequenz.
- Effekte auf die Atemmuskulatur sind nicht auszuschließen, würden aber nur bei prolongiertem Einsatz zu relevanter Hypoxie führen.
- Zu Risikogruppen, die großzügig medizinisch vorgestellt werden sollten, gehören psychisch erkrankte und intoxikierte Personen, Schwangere, Kinder und Device-Träger. Die Datenlage bezüglich des Risikos für diese Gruppen ist noch gering, und große randomisierte, kontrollierte Studien können aus ethischen Gründen nicht ohne Weiteres durchgeführt werden.
- Eine medizinische Überwachung ist bei beschwerdefreien Personen ohne Begleiterkrankungen nicht routinemäßig notwendig.
